# Economic burden and mental health of primary caregivers of perinatally HIV infected adolescents from Kilifi, Kenya

**DOI:** 10.1186/s12889-020-8435-0

**Published:** 2020-04-16

**Authors:** Patrick V. Katana, Amina Abubakar, Moses K. Nyongesa, Derrick Ssewanyana, Paul Mwangi, Charles R. Newton, Julie Jemutai

**Affiliations:** 1 Clinical Research (Neurosciences), KEMRI-Wellcome Trust Research Programme, Centre for Geographic Medicine Research (Coast), Box, Kilifi, PO Box 230-80108 Kenya; 2 Health Economics Research Unit, KEMRI-Wellcome Trust Research Programme, Centre for Geographic Medicine Research (Coast), Kilifi, Kenya; 3 Department of Public Health, Pwani University, Kilifi, Kenya; 4 Department of Psychiatry, University of Oxford, Oxford, UK; 5 Institute for Human Development, Aga Khan University, Nairobi, Kenya; 6 Child and Adolescent Studies, Utrecht University, Utrecht, Netherlands

**Keywords:** HIV/AIDs, Economic burden, Adolescents, Caregivers, Mental health, Depressive symptoms, Sub-Saharan Africa

## Abstract

**Background:**

Eighty per cent of perinatally HIV infected (PHI) adolescents live in sub-Saharan Africa (sSA), a setting also characterized by huge economic disparities. Caregiving is crucial to the management of chronic illness such as HIV/AIDS, but the economic costs and mental disorders borne by caregivers of PHI adolescents often go unnoticed. In this study, we evaluated economic costs, coping strategies and association between economic cost and mental health functioning of caregivers of perinatally HIV infected adolescents in Kilifi, Kenya.

**Methods:**

We used a cost of illness descriptive analysis approach to determine the economic burden and Patient Health Questionnaire (PHQ-9) to assess the caregivers’ mental health. Cross-sectional data were collected from 121 primary caregivers of PHI adolescents in Kilifi using a structured cost questionnaire. Economic costs (direct and indirect costs) were measured from primary caregivers’ perspective. We used descriptive statistics in reporting the results of this study.

**Results:**

Average monthly direct and indirect costs per primary caregiver was Ksh 2784.51 (USD 27.85). Key drivers of direct costs were transportation (66.5%) and medications (13.8%). Total monthly costs represented 28.8% of the reported caregiver monthly earnings. Majority of the caregivers borrowed resources to cope with high economic burden. About 10.7% of primary caregivers reported depressive symptoms. Caregivers with positive depression screen (PHQ-9 score ≥ 10) had high average monthly direct and indirect costs. However, this was not statistically different compared to costs incurred by caregivers who screened negative for depressive symptoms.

**Conclusion:**

Our study indicates that HIV/AIDS is associated with a significant economic burden for caregivers of adolescents living with HIV. Results underscore the need for developing economic empowerment and social support programmes that reduce the economic burden of caring for perinatally infected adolescent. These efforts may improve the mental health and quality of life of caregivers of adolescents living with HIV.

## Background

Despite the comprehensive implementation of strategies to prevent vertical transmission of HIV, millions of children and adolescents in sub-Saharan Africa (sSA) are still perinatally infected [1]. In 2017, 36.9 million people were living with HIV around the world [2]; 2.1 million of them were adolescents aged between 10 and 19 years, majority of whom reside in sSA [3, 4]. Over 80% of HIV infected adolescents acquired the infection through mother to child transmission [5]. Nevertheless, the advent and upsurge in the uptake of antiretroviral therapy (ART) in settings such as sSA has led to reduced HIV/AIDS-associated mortality and increased life expectancy [6, 7]. Most perinatally infected children are now surviving to adolescence and early adulthood [8].

Perinatally HIV infected (PHI) adolescents, like any other adolescents, require care which contributes to measurable costs on their caregivers [9]. The increased longevity due to improvement in treatment and care for people living with HIV comes with the long-term responsibility by primary caregivers and/or family members of caring for the HIV infected young person throughout their developmental span. This may require that the caregiver adapts to the costs and stresses of caregiving demands. Such costs often comprise both direct (costs due to resource use attributable to an illness such as consultation, drugs, tests and travel cost to hospital [10]) and indirect costs (costs incurred from reduction or halting work productivity due to work productivity [11]). In settings such as sSA where healthcare services are often out-of-pocket [12, 13], most of caregiving burden falls solely on primary caregivers of PHI adolescents. Of growing concern is that these caregivers are bearing more than their “fair share” of costs of caring for their adolescents. The costs seem to be hidden or overlooked as they are not well captured on official records; hence, the development of evidence-based policy is hindered.

Family expenditure on HIV/AIDS may increase substantially due to high level of long-term investment costs [14]. Although drugs for HIV/AIDS are free for patients at public health facilities in many parts of the world, some services involve a charge such as, hospital admission charges or charges for diagnosis of comorbid disease conditions [15]. Evidence suggest that caregivers are burdened by the costs of other components of care, such as medicine and treatment for opportunistic infections [16]. Additional costs include non-medical costs such as transport costs. The recurring expenses of accessing healthcare services and opportunity costs of time spent at the health facility for regular clinic visits and treatment of comorbid disease conditions may contribute to obstacles for service delivery, care and treatment of PHI adolescents and their families [17]. The costs incurred often have impoverishing effects on some households and hinder PHI adolescents from getting necessary care [18]. Furthermore, the costs may result in catastrophic health expenses in the context of fragile economic balance and low insurance coverage [19].

To meet the costs of illness, caregivers of PHI adolescents adopt coping strategies such as sale of assets and borrowing [20–22]. Such strategies can help to cope with immediate economic burden; however, they can also be potentially ‘risky’ for their future financial wellbeing. The selling of livelihood assets, for instance, reduces the caregiver’s/or family’s ability to generate future income [23, 24]. There exists a vast body of literature on economic costs of HIV in Kenya and other African countries. However, economic costs faced by caregivers of adolescents living with HIV have received relatively little attention.

Although several studies have focused on the mental health, wellbeing and quality of life of caregivers of PHI children and adolescents [25, 26], they did not simultaneously evaluate caregiver’s economic burden and its association to mental health. In this study, we set out to evaluate the economic burden, coping strategies and mental health of primary caregivers of perinatally HIV infected PHI adolescents from Kilifi, Kenya. Specifically, we assessed both direct and indirect costs incurred by primary caregivers in providing care to PHI adolescents. We also elicited their coping strategies in meeting the various demands for care and treatment for their PHI adolescents. Finally, we assessed the severity of caregivers’ depressive symptoms that may be associated with meeting the demands of care and treatment for their PHI adolescents.

## Method

### Study setting

We carried out this study at the Centre for Geography Medicine Research-Coast at the Kenya Medical Research Institute (CGMRC-KEMRI) located in Kilifi County, Coastal Kenya. It was part of a baseline phase in an on-going longitudinal larger Adolescent Health Outcome Study (AHOS) between January and July 2018.

Kilifi County had been estimated to have a population of 1.45 million by 2019, of whom, 61% are rural dwellers, and 22% are aged between 10 and 20 years [27, 28]. Kilifi is termed as a “moderate HIV county” with 4% HIV prevalence, and 6000 (19%) of people living with HIV are adolescents and youth aged 15–24 years [29]. About a third of Kilifi County is covered by Kilifi Health and Demographic Surveillance System (KHDSS) nested in the KEMRI-Wellcome Trust Research Programme. The economic activity within the KHDSS residence is mainly subsistence farming.

### Study design

This was a cross-sectional study nested in a larger adolescents’ health outcome study (AHOS). AHOS is an ongoing longitudinal study aimed at examining neurocognitive and mental health outcomes of 12–17 years old adolescents in the context of HIV. In this sub-study, we recruited primary caregivers accompanying PHI adolescents participating in the parent study. PHI caregivers in the parent study were recruited through consecutive sequential sampling from all families that attended HIV clinic days at eight HIV treatment and care clinics in Kilifi County until the targeted number was achieved. The selection of the eight clinics was done purposively based on distribution and client capacity of HIV specialized clinics within the KHDSS.

Primary caregivers of the adolescents attending a HIV clinic day were approached and briefed about the study during their waiting time at the HIV clinics. Informed consent was obtained from all the individual participants sampled in this sub-study. This included obtaining written parental or guardian consent as well as adolescents’ assent. Recruitment was done by a trained research assistant in liaison with local field worker and community health worker at the HIV treatment facilities. An adolescent living with HIV using anti-retroviral drugs (ARVs) has regular visits to the HIV specialized clinic for comprehensive care including anti-retroviral therapy (ART) refill, treatment for routine medical check-up, counselling services or nutritional advice (when recommended). Ethical approval to conduct this study was obtained from the Kenya Medical Research Institute Scientific and Ethics Review Unit (KEMRI/SERU/CGMR-C/084/3454) and permission from county government of Kilifi (HP/KCHS/VOL-VII/209).

### Data collection

We collected data using survey cost questionnaire/tool developed by adapting costing items from different existing tools [30, 31]. The items included information on direct and indirect costs incurred and coping cost strategies for PHI caregivers. The survey cost questionnaire was translated from English to Swahili and then back translated to ensure accuracy and coherence. We piloted the tool with 12 participants from an HIV specialized clinic in Kilifi prior to roll-out in this study. We further revised the questionnaire based on feedback and results from the pilot phase. Data from the pilot phase were not analysed in the sub-study. The final version of the tool was later designed in a tablet platform using Research Electronic Data Capture (REDCap) [32] and administered to primary caregivers of PHI adolescents.

### Sample size calculation

At the time of setting up the study, there was no integrated medical/health system data on exact number of adolescents in active follow-up. Since the parent study did not aim to do a population-level research on adolescents with HIV, we did not collect the data on number of adolescents attending the clinics. However, informally we had been advised that there were approximately 300 PHI adolescents in active follow-up across different clinics in Kilifi.

Sample size was calculated using Yamane’s formula [33], *n* = N/(1 + Ne2), as applied in previous similar costing study approaches [34]. In this formula, n is minimal required number of participants, N is the population size and e is the desired level of precision. We used population size of about 300 caregivers of perinatally HIV adolescents who are on active follow-up at HIV care centres in Kilifi and 7% precision level to compute a minimum sample size as follows.

$$ n=\frac{300}{1+300{(0.07)}^2}=121 $$

Assuming a 97% response rate, we estimated a sample size of 124 caregivers.

### Economic burden measures

We applied a cost of illness approach and measured direct costs, indirect costs and coping strategies from the primary caregivers’ perspective, i.e. we sought to elicit the economic costs incurred and paid for by the caregivers.

#### Direct cost

Average direct costs were measured by combining all the average out-of-pocket medical and non-medical costs for the PHI adolescent, caregiver and person accompanying PHI adolescent (other than the caregiver). These included administrative costs (registration and consultation); cost for diagnostic tests, medicine, other medical costs; food, accommodation/bed charges, and travel at the time of treatment visit. Besides the regular visits to the HIV specialized clinics, individuals living with HIV sometimes visit other healthcare providers for general treatment. In this study, the direct costs from regular visits and other healthcare provider visits were analysed. The average costs from these visits within the month of the interview was measured and assumed to be equivalent to the direct monthly costs due to HIV/AIDS in this study.

#### Indirect costs (productivity loss)

We examined the loss of productive working time by the caregiver due to illness of PHI adolescent. Productivity cost was defined as the inability to carry out normal daily activities (paid and unpaid), and their valuation in United States Dollar (USD). Normal activities were defined as formal and informal work carried out by the caregiver. To calculate productivity losses, the inability to work, were divided into absenteeism and presenteeism. Absenteeism was defined when the caregiver was unable to carry out normal daily activities at all due to adolescent illness. On the other hand, ‘*presenteeism*’ was defined when the caregiver reduced efficiency in work where he/she could work some hours, but not the whole day [35]. The summation of absenteeism and presenteeism was termed as ‘productivity loss’ and their monetary valuation was termed as ‘productivity cost’ in this study. Two aspects were considered: i) the days which losses were experienced; ii) the extent to which work efficiencies were affected in hours. Valuation of productivity losses in this study is presented as Gross Domestic Product (GDP) per capita of Kenya (USD 4.11) in a day, according to the 2017 World Bank report [36]. Valuation using per capita is preferred because this approach values time loss for the rich and the poor people by an average of the whole society [30].

### Mental health assessment

The 9-item Patient Health Questionnaire (PHQ-9) [37] was also administered to the caregivers to assess their mental health functioning. The PHQ-9 is a screener and provides an indicator of depressive symptoms severity. The measure is scored on a 4-point Likert scale from ‘0’ (not at all) to ‘3’ (nearly every day) with total scores ranging from 0 to 27. As a measure of severity of depressive symptoms, minimal and mild symptoms of depression are interpreted as PHQ-9 total score of 0–4 and 5–9, minor and moderate depressive symptoms equate to PHQ-9 total score of 10–14 and 15–19 respectively, and severe depressive symptoms equate to PHQ-9 scores of 20 or more. In this study, we used a cut off of ≥10 to indicate a positive screen for depressive symptoms. This cut-off maximizes the sensitivity and specificity in a study conducted in sSA [38]. PHQ-9 has proved to be reliable on primary healthcare setting with a Cronbach alpha of 0.86 from its original validation [37]. Data from the PHQ-9 and socio-demographic data were collected from the parent study.

### Statistical analysis

Descriptive statistics were used to present caregiver’s socio-demographic characteristics, caregiving costs, coping strategies and mental health. Means and median on continuous data and proportions on categorical data are summarized. Mean (average) costs were mainly reported because total cost, which is relevant and vital to society, can be directly estimated from mean cost [39–41]. However, we also used the Shapiro-Wilk test to test for normality. Median values were also reported, and Mann-Whitney U test was used where applicable. Data were analysed using R statistical software package, version 3.4.3 [42].

## Results

A total of 124 caregivers of PHI adolescents were approached to participate in this sub-study. Two caregivers did not seem to comprehend the questions and one had an adolescent who had refused to take medicine for a period of 2 years. These caregivers were excluded from the study. Consequently, analysis involved responses of 121 caregivers who successfully completed the questionnaire. Table 1 highlights caregiver’s socio-demographic characteristics. Most of the caregivers were female (86.0%) and biological parents of the adolescents (62.8%). The mean age of the caregivers was 46 years (SD = 12.42) and 41 (33.9%) had no formal education. Majority of the caregivers (98.3%) were not in employed professional occupations and were mainly in farming (38.0%) and small-scale business ventures (39.7%). Less than a quarter of the caregivers (9.1%) reported to have an active subscription with the National Hospital Insurance Fund (NHIF); a government-run health insurance coverage system in Kenya. This insurance cover in Kenya partly absorbs some of the costs but not full HIV/AIDS treatment [43]. However, none of the caregivers registered with NHIF reported to use this cover to seek healthcare for their adolescents living with HIV.

**Table 1:** Caregivers’ socio demographic characteristics (*N* = 121)

Characteristics	Frequency (n)	Percent (%)
Caregiver		
Biological Mother	64	52.9
Biological Father	12	9.9
Relative	45	37.2
Sex		
Female	104	86.0
Male	17	14.0
Age (years)		
< 20	3	2.5
20–29	16	13.2
30–39	23	19.0
40–49	40	33.1
Above 49	31	25.6
Unknown	8	6.6
Caregiver’s Education level		
No education	41	33.9
Partial primary	36	29.8
Primary graduate	25	20.7
Partial high school	6	5.0
High school graduate	10	8.3
Tertiary	3	2.5
Marital status		
Never married	16	13.2
Married	68	56.2
Separated	4	3.3
Divorced	0	0.0
Widowed	33	27.3
Occupation		
Farmer	46	38.0
Trader/Business	48	39.7
Casual laborer	10	8.3
Professional	2	1.7
Other occupation	15	12.4
Health insurance		
Yes	11	9.1
No	110	90.9

### Economic costs

The average monthly cost per caregiver was Kenyan shilling (Ksh) 2785.51 equivalent to United States Dollar (USD) 27.85, with direct costs being Ksh 1671.98 (USD 16.72) and indirect costs being Ksh 1112.5 (USD 11.13) (Table 2). Total cost incurred by participants in this study was Ksh 336,926 (USD 3369.26). For both average and total costs, direct costs constituted 60.05% and indirect costs 39.95%. The main key drivers of direct costs were transportation (66.5%) and medications (13.8%).

**Table 2:** Economic cost to caregiving for perinatally HIV infected adolescents

Cost component	Mean monthly costs per caregiver		Median USD [IQR]	Proportion of total costs^c^ (%)
	Kenyan shilling (SD)	USD^a^ (SD)		
Direct costs				
Direct medical cost				
Administrative costs	39.34 (95.74)	0.39 (0.96)	0.0 [0.0–0.2]	1.41
Diagnostics	53.72 (168.73)	0.54 (1.69)	0.0 [0.0–0.0]	1.93
Medicine	231.14 (427.21)	2.31 (4.27)	0.0 [0.0–3.0]	8.31
Other medical costs	0.00 (0.00)	0.0 (0.00)	0.0 [0.0–0.0]	0.00
Subtotal	324.20 (565.06)	3.24 (5.65)	0.5 [0.0–4.0]	11.65
Direct non-medical cost				
Travel	1111.49 (931.43)	11.11 (9.31)	8.0 [4.0–16.0]	39.92
Food	233.79 (270.36)	2.34 (2.70)	1.6 [0.0–3.7]	8.40
Bed charges	2.50 (27.39)	0.03 (0.27)	0.0 [0.0–0.0]	0.08
Subtotal	1347.78 (1104.25)	13.48 (11.04)	9.5 [5.6–20.1]	48.40
Total direct costs	1671.98 (1221.76)	16.72 (12.22)	12.0 [7.5–24.3]	60.05
Indirect costs (Productivity costs)^b^				
Absenteeism	733.69 (725.98)	7.34 (7.26)	4.1 [4.1–8.2]	26.35
Presenteeism	378.84 (122.83)	3.79 (1.23)	3.8 [3.0–4.5]	13.60
Total indirect costs	1112.53 (753.05)	11.13 (7.53)	8.8 [7.1–13.3]	39.95
Grand Total Cost	2784.51 (1492.33)	27.85 (14.92)	25.5 [16.4–37.6]	100.00

^a^USD – United States Dollar; 1.00USD equivalent to Kenyan shilling 100.4 (Central Bank of Kenya monthly interbank exchange rate, August 2018)

^b^ Absenteeism (days caregiver was completely unable to work), Presenteeism (The decrement in performance associated with the caregiver remaining at work while impaired by adolescent health problems, hours lost converted to days). Valuation was done using per capita GDP in 2017 (Ksh 411/day) (World Bank data [36])

^c^ Percentage computed by diving the total cost from the cost component item by the grant total cost and multiplying the result by 100

IQR-Interquartile range

The study found that the average number of days a caregiver was completely unable to carry out normal activities due to illness of PHI adolescent in a month (absenteeism) was 1.78. The average number of days lost due to sub-optimal work efficiencies was 0.92. Therefore, average productivity loss in a period of 1 month was a total 2.7 days. Average productivity costs were calculated by multiplying average days lost by the caregiver in a month with per capita GDP in Kenya as of the year 2017. Thus, average productivity costs due to caregiving in a month were Ksh 1112.53 (USD 11.13) (Table 2).

Average total costs were calculated by summing direct costs and productivity costs (indirect costs) due to HIV/AIDS in a monthly period. Thus, the average total monthly costs per caregiver was Ksh 2784.51 (USD 27.85), which was 28.8% of the average caregiver’s monthly income.

### Coping strategies

**Fig. 1:**
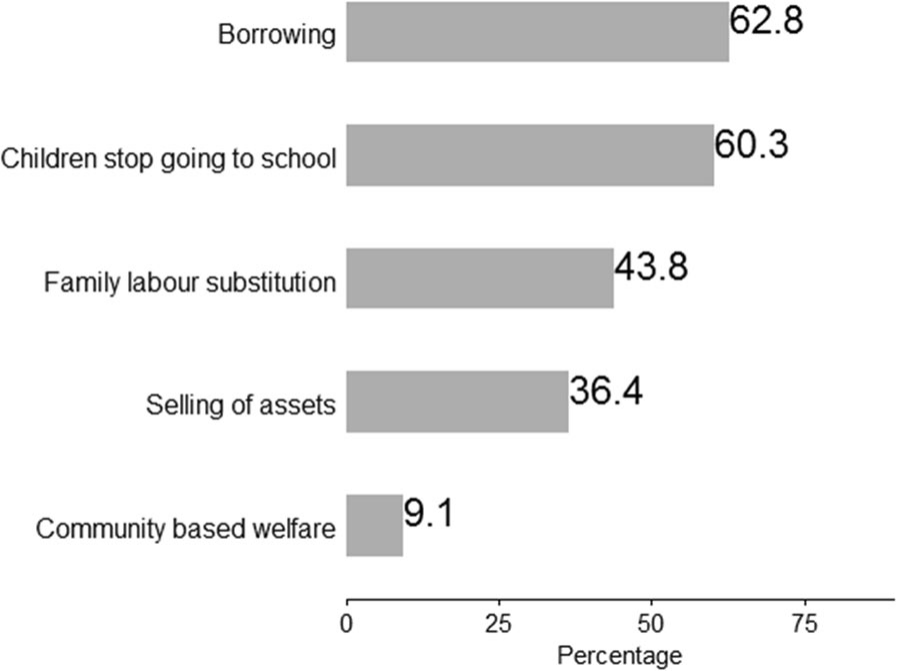
Coping strategies

Adolescents’ caregivers were asked on their coping behaviours that have an effect on minimizing the impact of costs on caregiver’s families due to illness. We also inquired about any amount of money raised from different sources to support treatment over a period of 4 months. Caregivers indicated use of combined coping strategies, as shown in Fig. 1.

### Caregiving costing and mental health outcomes.

A total of 44 (36.4%), 64 (52.9%), 11 (9.1%), 1 (0.8%) and 1 (0.8%) caregivers had minimal, mild, minor, moderate and severe depressive symptoms respectively. Based on a cut-off score of ≥10 on the PHQ-9, thirteen (10.7%) primary caregivers of PHI adolescents screened positive for depressive symptoms (Table 3). It was observed that direct, indirect and total costs of caregiving for PHI adolescents were higher among caregivers with a positive screen for depressive symptoms compared to those without depression symptoms.

**Table 3:** Caregiving costs in USD per caregiver and mental health outcomes

Cost categories	No depressive symptoms (score < 10) ***n*** = 108		Depressive symptoms (score ≥ 10) ***n*** = 13		
	***Mean costs*** ***(SD)***	***Median costs [IQR]***	***Mean costs*** ***(SD)***	***Median costs [IQR]***	P-value*
Direct Costs	16.32 (12.19)	11.6 [4.9–13.5]	19.46 (12.66)	19.2 [15.9–21.3]	0.33
Indirect Costs	10.76 (7.44)	8.7 [5.1–12.0]	13.84 (7.99)	14.3 [12.0–17.5]	0.09
Total Costs	27.21 (14.87)	23.9 [16.7–30.1]	34.01 (14.62)	40.2 [18.1–47.0]	0.11

*Mann-Whitney tests on non-normal cost data

*USD* United States Dollars, *SD* Standard deviation, *IQR* Interquartile range*.*

## Discussion

This study examined the economic burden and mental health of primary caregivers of perinatally HIV infected (PHI) adolescents, using data from 121 primary caregivers. This is especially important, as limited research has been done around economic burden and mental health of caregivers of PHI adolescents from sSA, yet sSA carries the biggest burden of HIV [44]. We found that caregivers of PHI adolescents experienced a high economic burden, and in some cases, applied catastrophic coping strategies, also known as risk or stress level coping strategies. Additionally, we observed that caregivers with depressive symptoms had a higher economic cost burden compared to those without.

The economic burden for the caregivers interviewed was high, representing approximately a quarter of their monthly income. This economic burden is likely to contribute to lowered quality of life for the caregiver [45–47] as funds for other important family expenditure and investment may become scarce. The highest burden was associated with travel costs to attend clinic appointments and then followed by costs of purchasing drugs largely for opportunistic infections and other health conditions. These results have a two-fold implication on policy planning for health care. First, the need to avail paediatric HIV care closer to community settings will be important to reduce the travel costs to patients and their families. Secondly, other required treatment plans/medication should be given freely alongside ARVs to reduce the caregiver costs and expenditures on purchasing of drugs.

Productivity loss is an important economic burden in the context of HIV and surprisingly few studies have investigated productivity loss in sub-Saharan Africa. We noted there were studies related to productivity losses due to HIV/AIDS on adult population in other countries. A study in Nepal [30] reported lower but comparable productivity costs. Higher productivity costs in our study might be due to the following reasons: i) the specialised HIV clinic is located far from most care recipients, ii) poverty among most households thereby experiencing high levels of vulnerability and low accessibility to basic necessities, and iii) worse treatment and care outcomes among the PHI adolescents or/and their caregivers in our study setting. Hence it leads to loss of productivity by the caregiver since much time is spent on taking care of a sick adolescent rather than performing normal income-generating activities.

It is worth noting that the government of Kenya gives a monthly transfer cash payment of Ksh 2000 (USD 20) to primary caregivers of Orphans and Vulnerable Children (OVC) [48]. This programme was set up as a way to provide a financial buffer for caregivers of OVC. However, our findings possibly indicate potential weakness of this public health intervention in Kenya. First, in this study, most caregivers fulfilled the criteria to receive the cash transfer, but only three caregivers reported the cash transfer as their source of income. We hypothesize that the low uptake of the cash transfer may result from low awareness or bottleneck bureaucratic measures one must undertake to access to the fund. There is an urgent need to understand this barrier and propose measures that will ensure that equity is maintained in the distribution of support funds to caregivers of orphans and vulnerable children.

Caregivers of PHI adolescents in our setting applied various coping strategies to meet the need of those under their care. However, some of the reported coping mechanisms are detrimental to the economic wellbeing of the family. For instance, the sale of family assets or borrowing (especially with a high-interest rate) risk long-term economic hardship for the caregiver and/or family [23, 24]. Moreover, children missing out on school attendance affects future economic wellbeing of the adolescents and their family. These results provide further impetus for the development of more economic empowerment programmes for caregivers of PHI adolescents to save the family from spiralling into poverty.

Nearly 11% of these primary caregivers screened positive for depressive symptoms. Even though not statistically significant, high direct costs and indirect costs (productivity costs) burden were observed in caregivers with depressive symptoms compared to those without. A similar trend was observed by Harmanci and Cetinkaya [49], who reported a significant association between mental health and economic burden. We had only 13 out of 124 people screen positive for depression while in the study by Harmanci and Cetinkaya, their number of screen positives were 90 out of 104. Future work with larger sample size need to examine these associations in greater detail.

The results of this study should be interpreted within the context of some limitations. We undertook a cross-sectional study with a small sample size; we cannot assume that the economic burden applies to the larger caregiver population of adolescents living with HIV in Kenya. In addition, our sample is derived from a rural coastal Kenyan setting which is not representative of all HIV affected households/settings of Kenya. In addition, the data were derived from self-reported caregivers who needed to recall their past caregiving costs. Therefore, we cannot rule out the possibility of response and memory bias.

## Conclusions

Although ARV drugs have been fully subsidized in HIV comprehensive care centres in Kenya, caregivers of PHI adolescents are still incurring considerable expenses (relative to their incomes) when seeking care for their child. These high economic burdens may potentially contribute to a vicious cycle of poverty, poor mental health for the caregivers and reduced educational opportunities for adolescents. It is therefore recommended that a further review on the financial support for families caring for perinatally infected adolescents be undertaken to ensure long-term health of the affected adolescent is guaranteed and consequently better economic growth from productive individuals and households. Likewise, we recommend a longitudinal study for caregiving costs and mental health, including a control group to compare different domains under investigation.

## Data Availability

Anyone interested in accessing the data reported in this article is free to write to the Data Governance Committee of the KEMRI Wellcome Trust Research Programme who will review the application and advise as appropriate and ensure that users are compatible with the consent obtained from participants for data collection. Requests can be sent to the coordinator of the Data Governance Committee using the following email: dgc@kemri-wellcome.org.

## Abbreviations

AHOS: Adolescent health outcome study
AIDS: Acquired immune deficiency syndrome
ART: Antiretroviral therapy
ARV: Antiretroviral
CGMRC-KEMRI: Centre for geography medicine research-coast at the Kenya medical research institute
GDP: Gross domestic product
HIV: Human immune virus
KHDSS: Kilifi health and demographic surveillance system
NHIF: National hospital insurance fund
OVC: Orphans and vulnerable children
PHI: Perinatally HIV infected
PHQ: Patient health questionnaire
REDCap: Research electronic data capture
USD: United States dollar
